# Prevalence of frailty in Indonesia: a systematic review and meta-analysis

**DOI:** 10.1186/s12877-023-04468-y

**Published:** 2023-11-27

**Authors:** Anung Ahadi Pradana, Huei-Ling Chiu, Chen-Ju Lin, Shu-Chun Lee

**Affiliations:** 1 STIKes Mitra Keluarga, Bekasi-Indonesia, Indonesia; 2 International PhD Program in Gerontology and Long-Term Care, College of Nursing, Taipei Medical University, Taipei, Taiwan; 3 School of Gerontology and Long-Term Care, College of Nursing, Taipei Medical University, Taipei, Taiwan; 4 Department of Rehabilitation Medicine, Taipei Tzu Chi Hospital, Buddhist Tzu Chi Medical Foundation, New Taipei, Taiwan

**Keywords:** Frailty, Indonesia, Older adults, Meta analysis, Prefrailty, Prevalence

## Abstract

**Background:**

Frailty increases the risks of hospitalization, injury, fall, psychological disorders, and death in older adults. Accurate estimation of the prevalence of frailty is crucial for promoting health in these individuals. Therefore, this study was conducted to estimate the prevalence of frailty and prefrailty in older adults residing in Indonesia.

**Methods:**

In accordance with the Preferred Reporting Items for Systematic Reviews and Meta-Analysis guidelines, six electronic databases were searched (without any language restriction) for relevant articles from inception to February 2023. Studies on the prevalence of frailty and prefrailty in older adults (age ≥ 60 years) residing in Indonesia were included in the analysis. A random-effects model was selected a priori because of the expected high degree of heterogeneity in the study, followed by sensitivity analysis, subgroup analysis, and meta-regression. The protocol of this review study was registered in the PROSPERO database (CRD42022381132).

**Results:**

A total of 79 studies were identified, of which 20 were finally included in the analysis. The pooled prevalence of frailty and prefrailty in older adults in Indonesia was 26.8% and 55.5%, respectively. The pooled prevalence of frailty and prefrailty was 37.9% and 44.8% in nursing homes, 26.3% and 61.4% in hospitals, and 21.1% and 59.6% in community settings, respectively. Furthermore, the pooled prevalence of frailty and prefrailty was 21.6% and 64.3%, 18.7% and 62%, and 27.8% and 59.8% in studies using the Frailty Index-40, FRAIL, and Fried Frailty Phenotype questionnaires, respectively. However, the parameters did not vary significantly across measurement tools or study settings. Publication bias was not detected while the year of data collection influenced the heterogeneity between the studies.

**Conclusions:**

To the best of our knowledge, this study is the first meta-analysis to report the prevalence of frailty and prefrailty in older adults residing in Indonesia. The gradual increase in the number of older adults with frailty or prefrailty in Indonesia is concerning. Therefore, the government, private sectors, health-care professionals, and the community must jointly design effective strategies and policies to address this problem.

## Background

Frailty, a geriatric symptom, reduces older adults’ stress resistance and disturbs the body’s homeostatic balance. In addition, it reduces resistance to various harmful agents entering the body, elevates the risks of injury and immobility, and increases the rates of hospitalization and mortality in older adults [1]. Frailty is associated with several factors, such as age, sex, low education level, cohabitation (with family), comorbidities, polypharmacy, social isolation (limited engagement in activities outside the home), nonfunctional ambulation, and malnutrition [2].

The increase in the aging population worldwide is proportional to the incidence of degenerative and frailty-related diseases [3]. The estimated prevalence of frailty in the global population of older adults aged > 85 years is 25%–50%. However, the prevalence varies widely (range, 3%–47.2%) depending on age and sex. The prevalence of frailty varies across countries, with studies reporting the following prevalence percentages: China, 3.9%; Turkey, 39.2%; and Cuba, 51.4%. The prevalence of prefrailty also varies across countries, with studies reporting the following percentages: Tanzania, 13.4%; Turkey, 43.3%; and Brazil, 71.6% [2, 4]. In Singapore, which is the closest country to Indonesia, the prevalence of frailty in community-dwelling older adults is 5.7%–24.5% [5, 6]. In the countries neighboring Indonesia, such as Malaysia and Thailand, the prevalence of frailty in community-dwelling older adults is 8.9%–15.9% [7, 8] and 8.7%–22.1% [9, 10], respectively.

The modern world is facing an unprecedented challenge of aging populations. At least two-thirds of the global population of older adults reside in low-to-middle-income countries; the rate of population aging in these countries is expected to exceed the rate in high-income countries after 2025 [3]. The proportion of older adults in Indonesia increased to 10.7% (an increase of 5.2% over the last five decades) in 2020 and is projected to reach 19.9% by 2045 [11]. Concurrently, older adults’ health problems (e.g., frailty) have been increasing.

In Indonesia, the increase in the number and life expectancy of older adults has resulted in problems such as an increase in the number of older adults without any income who are forced to depend on others. The morbidity rate of such older adults in the community has been reported to be 25.05% [12]. In addition, the frailty condition experienced by older adults can exacerbate this burden on the community [13].

Indonesian have culturally strong family characteristics, this indirectly strengthens the social bond between older adults and their relatives. Social capital is one of the most important factors in strengthening biological and psychological perspectives in relation to the complexities of older adults’ health [5]. In the structure of Indonesian society, older adults are often considered a burden on society because they experience a decrease in intrinsic capacity such as physical, mental and cognitive capacity, thereby hampering functional abilities. The increasing number of vulnerable older adults can indirectly result in negative social and economic impacts on society [2]. Negative impacts that can occur include low community productivity, financial disruption, caregiver burden, and high costs of health services which could lead to a huge problem on the state [6].

Therefore, accurate country-specific prevalence data are essential for the implementation of priority interventions by the government and health workers to identify, manage, and prevent problems of related to the older population. To the best of our knowledge, no study has explored the prevalence of frailty in Indonesia. Therefore, this systematic review and meta-analysis was conducted to clarify the prevalence of frailty and prefrailty in older adults residing in Indonesia. Our findings regarding the overall prevalence of prefrailty and frailty in Indonesia may guide policies for reducing frailty-related problems in older adults.

## Methods

Relevant articles were systematically identified in accordance with the Preferred Reporting Items for Systematic Reviews and Meta-Analysis (PRISMA) guidelines [14]. The protocol of this review study was registered in the PROSPERO database (CRD42022381132).

### Data sources and search strategy

PubMed, Cochrane Library, CINAHL, EMBASE, Ovid, and GARUDA web were searched (without any language restriction) for relevant articles from inception to November 2023. GARUDA web is an Indonesian science database containing Indonesian articles that have been published in Indonesian journals. The following Medical Subjective Heading terms were searched with an explosion function (if available): “frailty,” “prevalence,” or “epidemiology” and “Indonesia.” Articles containing a combination of the aforementioned keywords in the title and main text were retrieved. The year of publication was not limited because few studies have focused on frailty, particularly in Indonesia. No language restriction was imposed to avoid excluding articles likely to contribute positively to our analysis.

### Selection criteria

The authors used the Population Intervention Comparison Outcomes (PICO) framework [15] as the basis for the method of the selection criteria as follows: (1) Population: the population was older adults aged > 60 years; (2) Intervention and Comparison were not included due to the prevalence study; and (3) Outcome: the reported outcome consists of frailty and/or prefrailty parameters. Studies on the prevalence of frailty and/or prefrailty in older adults (age ≥ 60 years) residing in the community, nursing homes or clinics, and hospitals in Indonesia were included. Studies involving older adults with comorbidities, and articles that were theses, abstract proceedings, literature reviews, editorials, and letters to the editor were excluded from our analysis. Two reviewers (AAP and SCL) screened the relevant articles on the basis of the inclusion and exclusion criteria.

### Data extraction and methodological quality assessment

The following data were extracted by one reviewer (AAP) and independently confirmed by another reviewer (SCL): the participants (sample size and mean age), measurement tools (Fried frailty phenotype, Frailty Index-40 (FI-40) questionnaire, FRAIL questionnaire, Edmonton Frail Scale (EFS), and Survey of Health, and Ageing and Retirement in Europe (SHARE) frailty instrument], study settings (community, hospital, and nursing home), provincial locations, and outcomes (prevalence of frailty or prefrailty). Any disagreement between the two authors was resolved by a third author (HLC) through discussion.

Three reviewers (AAP, SCL, and HLC) critically appraised the included articles by using the risk of bias in nonrandomized studies of exposures (ROBINS-E) visualization tool, which can be used to evaluate the quality of study methodology across seven domains. They used the following seven domains of bias in the ROBINS-E tool: (1) risk of bias due to confounding, (2) risk of bias arising from the measurement of the exposure, (3) risk of bias in the selection of participants for the study, (4) risk of bias due to post-exposure interventions, (5) risk of bias due to missing data, (6) risk of bias arising from the measurement of the outcome, and (7) risk of bias in the selection of the reported result [16]. The ROBINS-E is designed primarily for use in systematic reviews, where it is used to measure the strength of evidence to determine the presence or nature of the potential effect of an exposure on an outcome.

### Frailty measurement tools

Several measurement tools were used in the included studies: Fried frailty phenotype, FRAIL questionnaire, FI-40 questionnaire, EFS, and SHARE frailty instrument. Of them, the FRAIL and Fried scales mainly focus on the physical component of frailty, separating it from disability and comorbidity, namely, the ‘phenotype of frailty’ model, whereas the SHARE frailty instrument is based on conditions or disabilities, and it tends to emphasize the number rather than the nature of deficits, namely, the ‘accumulation of deficits’ model [17, 18]. The Fried frailty phenotype is a standardized protocol that categorizes older adults as having frailty based on five characteristics: (1) unintentional weight loss, (2) weakness, (3) exhaustion, (4) slowness, and (5) low activity level. Those with no frailty characteristics are considered robust, whereas those with one or two characteristics are hypothesized to be in an intermediate, possibly prefrail, stage clinically [19].

The Frail Scale comprises 5 questions (yes–no answer), and based on their total score, patients are categorized as robust (0 points), prefrail (1–2 points), and frail (> 3 points). The scale assesses the presence of fatigue, muscle resistance, aerobic capacity, disease burden, and weight loss [20]. The FI-40 questionnaire is a self-report measurement tool assessing symptoms, illness, health attitudes, and changes in function in community-dwelling older adults [21]. The EFS can be used by non-geriatricians. It comprises 10 domains; the maximum score is 17 and represents the highest level of frailty [22]. The SHARE frailty instrument is a special 4-criterion tool developed to help general practitioners assess frailty [23], but it requires further evaluation in larger studies for its widespread use in all settings [24].

### Data analysis

A meta-analysis was performed after sufficient homogeneity was achieved across the included studies. *I*^2^ statistics were calculated to evaluate homogeneity across the studies; an *I*^2^ value of < 50% indicated low heterogeneity [25]. The transition rates of the included studies were pooled through a random-effects meta-analysis. A random-effects model was selected a priori because of the expected high degree of heterogeneity in the study populations, settings, and outcomes [25]. Sensitivity analysis was conducted to determine whether the study with a high risk of bias significantly affected the results of this study.

Publication bias was evaluated graphically by generating funnel plots to determine the level of heterogeneity of the included studies. An asymmetric funnel indicated a publication bias, which was statistically verified using Begg’s rank correlation test and Egger’s linear regression test. The Comprehensive Meta-Analysis tool was used for all analyses. Data are presented in terms of percentages and 95% confidence intervals (CIs). To obtain the pooled estimates of frailty and prefrailty prevalence, a subgroup meta-analysis was performed after the studies were stratified by their settings and measurement tools; the meta-regression analysis was performed considering p < 0.005 to determine whether covariates such as mean age, sample size, and data collection years can explain the between-study heterogeneity.

## Results

### Characteristics of the included studies

**Fig. 1:**
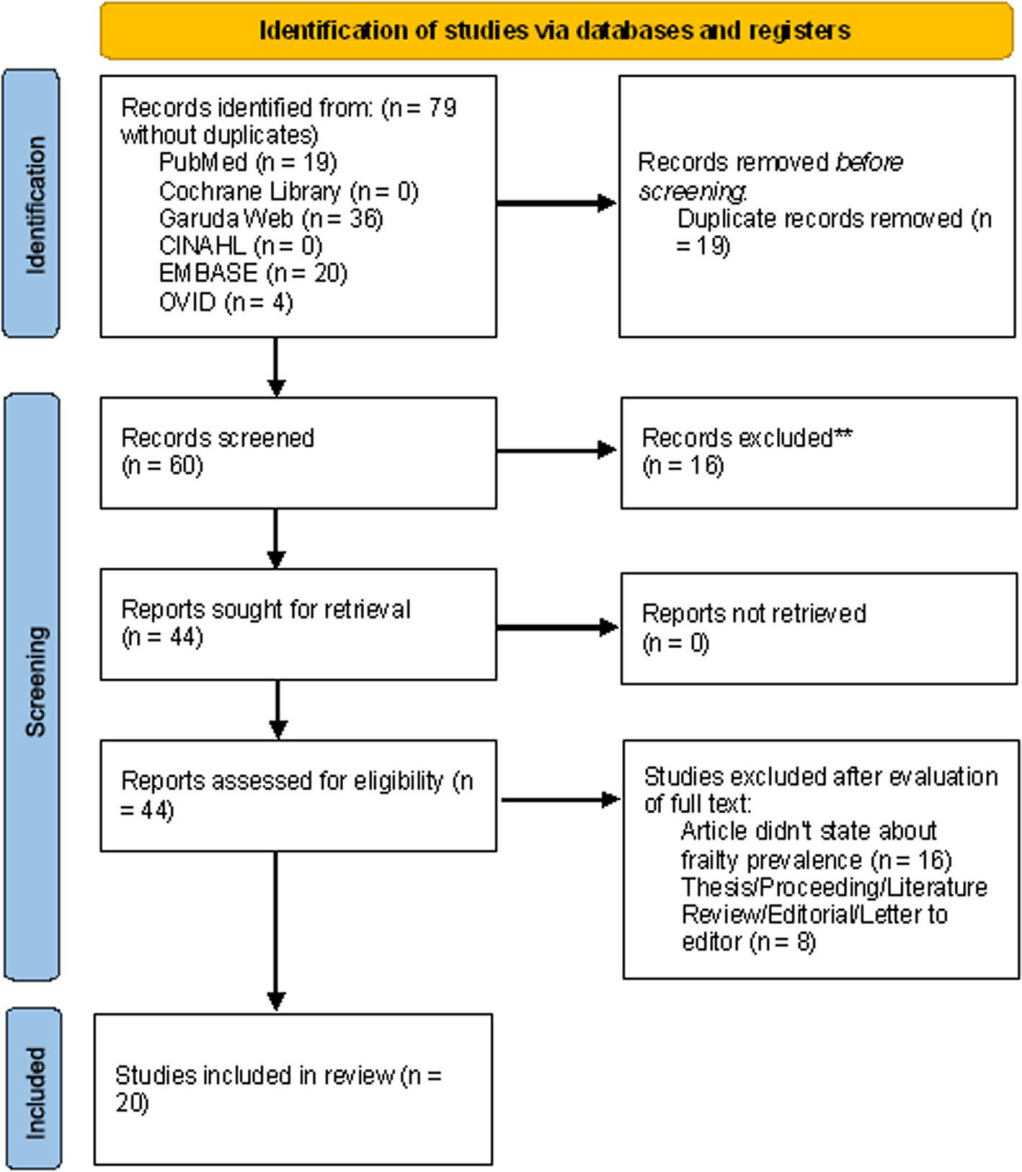
PRISMA flowchart

The literature search returned 79 articles, of which 20 articles (total participants = 6719; sample size = 27–2630) satisfied the inclusion criteria (Fig. 1). Seventeen articles were written in English, whereas three articles were in Indonesian. Frailty was assessed using the Fried Frailty Phenotype questionnaire in 12 studies (60%); the fatigue, resistance, aerobic, illness, and loss of weight (FRAIL) questionnaire in 3 studies (15%); the Frailty Index 40 (FI-40) questionnaire in 3 studies (15%); the Edmonton Frail Scale (EFS) in 1 study (5%); and the Survey of Health, Ageing and Retirement in Europe (SHARE) database in 1 study (5%). In terms of study settings, 8 (40%) studies were conducted in community settings, 7 (35%) were conducted in hospitals, and 5 (25%) were conducted in nursing homes. Fifteen articles reported the prevalence of both frailty and prefrailty in older adults, whereas five studies reported the prevalence of only frailty (Table 1).

**Table 1:** Characteristics of included studies

No	References	Participants	Measurement tool	Setting	Prevalence (%)
1	Aprianta et al., 2020 [26]	**Sample size:** 30 **Sex:** Men, 8 (26.67%) Women, 22 (73.33%) **Age (mean):** 73.73 years	Fried Frailty Phenotype questionnaire	Nursing home	**Frailty,** 43.30 **Prefrailty,** 56.70
2	Arjuna et al., 2017 [27]	**Sample size:** 527 **Sex:** Men, 215 (40.80%) Women, 312 (59.20%) **Age (mean ± SD):** 74 ± 7 years	FRAIL questionnaire	Community	**Frailty,** 13
3	Darwis & Safei, 2022 [28]	**Sample size:** 27 **Sex:** Men, 8 (29.63%) Women, 19 (70.37%) **Age (mean ± SD):** 73.15 ± 8 years	Edmonton Frail Scale	Nursing home	**Frailty,** 44.40
4	Faizah et al., 2022 [29]	**Sample size:** 113 **Sex:** Men, 16 (14.16%) Women, 97 (85.84%) **Age (mean):** 66.62 years	Fried Frailty Phenotype questionnaire	Community	**Frailty,** 46.90
5	Handajani et al., 2015 [30]	**Sample size:** 138 **Sex:** Men, 67 (48.55%) Women, 71 (51.45%) **Age (mean ± SD):** 71.8 ± 7.9 years	SHARE database	Nursing home	**Frailty,** 52.20 **Prefrailty,** 30.40
6	Jayadi et al., 2020 [31]	**Sample size:** 77 **Sex:** Men, 29 (37.66%) Women, 48 (62.34%) **Age (mean ± SD):** 68.16 ± 6.45 years	Fried Frailty Phenotype questionnaire	Hospital	**Frailty,** 24.70 **Prefrailty,** 44.20
7	Laksmi et al., 2019 [32]	**Sample size:** 120 **Sex:** Men, 46 (38.33%) Women, 74 (61.67%) **Age:** Unreported	Fried Frailty Phenotype questionnaire	Hospital	**Frailty,** 29.17 **Prefrailty,** 58.33
8	Ngestiningsih et al., 2020 [33]	**Sample size:** 70 **Sex:** Women, 70 (100%) **Age (mean):** 65.96 years	Fried Frailty Phenotype questionnaire	Community	**Frailty,** 4.30 **Prefrailty,** 70
9	Ngestiningsih et al., 2021 [34]	**Sample size:** 27 **Sex:** Women, 27 (100%) **Age (mean):** 67.93 years	Fried Frailty Phenotype questionnaire	Community	**Frailty,** 11.11 **Prefrailty,** 59.26
10	Pengpid & Peltzer, 2019 [35]	**Sample size:** 2630 **Sex:** Men, 1300 (49.43%) Women, 1330 (50.57%) **Age (median):** 66 years	Fried Frailty Phenotype questionnaire	Community	**Frailty,** 8.10 **Prefrailty,** 61.60
11	Permatasari et al., 2018 [36]	**Sample size:** 140 **Sex:** Men, 60 (42.86%) Women, 80 (57.14%) **Age:** Unreported	FRAIL questionnaire	Hospital	**Frailty,** 27.10 **Prefrailty,** 32.90
12	Rahmadani et al., 2018 [37]	**Sample size:** 72 **Sex:** Men, 23 (31.94%) Women, 49 (68.06%) **Age:** Unreported	FI-40 questionnaire	Nursing home	**Frailty,** 9.70 **Prefrailty,** 58.30
13	Rensa et al., 2019 [38]	**Sample size:** 325 **Sex:** Women, 325 (100%) **Age (median):** 67 years	Fried Frailty Phenotype questionnaire	Community	**Frailty,** 24 **Prefrailty,** 63.40
14	Rizka et al., 2021 [39]	**Sample size:** 214 **Sex:** Men, 62 (28.97%) Women, 152 (71.03%) **Age (mean ± SD):** 73.7 ± 4.3 years	Fried Frailty Phenotype questionnaire	Nursing home	**Frailty,** 46.70 **Prefrailty,** 51.30
15	Setiati et al., 2019 [40]	**Sample size:** 448 **Sex:** Men, 180 (40.18%) Women 268 (59.82%) **Age (mean ± SD):** 72.9 ± 5.9 years	FI-40 questionnaire	Hospital	**Frailty,** 25.20 **Prefrailty,** 61.6
16	Setiati et al., 2021 [41]	**Sample size:** 908 **Sex:** Men, 438 (48.24%) Women, 470 (51.76%) **Age:** Unreported	FRAIL questionnaire	Hospital	**Frailty,** 18.70 **Prefrailty,** 66.20
17	Seto et al., 2015 [42]	**Sample size:** 269 **Sex:** Men, 106 (39.4%) Women, 163 (60.6%) **Age (median):** 72 years	FI-40 questionnaire	Hospital	**Frailty,** 25.30 **Prefrailty,** 71
18	Sunarti & Hariyanti, 2018 [43]	**Sample size:** 212 **Sex:** Men, 46 (21.7%) Women, 166 (78.3%) **Age:** Unreported	Fried Frailty Phenotype questionnaire	Community	**Frailty,** 35.40 **Prefrailty,** 47.20
19	Triguna et al., 2021 [44]	**Sample size:** 62 **Sex:** Men, 62 (100%) **Age (mean ± SD):** 69.26 ± 7.11 years	Fried Frailty Phenotype questionnaire	Community	**Frailty,** 50
20	Widajanti et al., 2020 [45]	**Sample size:** 308 **Sex:** Men, 78 (25.3%) Women, 230 (74.7%) **Age (median):** 63 years	Fried Frailty Phenotype questionnaire	Hospital	**Frailty,** 36.70

*FI-40* Frailty Index 40, *FRAIL* fatigue, resistance, aerobic, illness, and loss of weight, *SD* standard deviation, *SHARE* Survey of Health, Ageing and Retirement in Europe

**Fig. 2:**
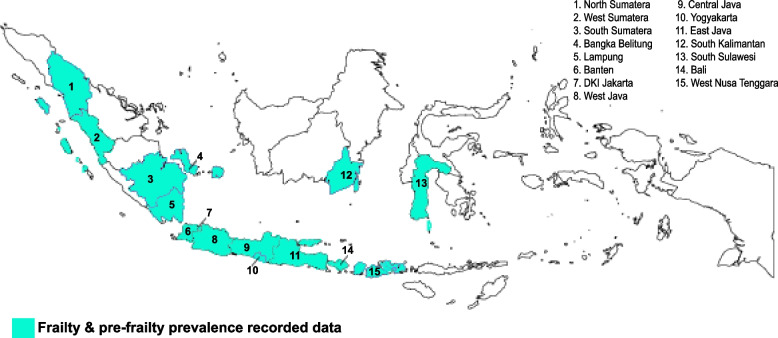
Overview of provincial locations identified in included articles

The included studies covered 15 of 38 provinces in Indonesia (Fig. 2), representing the country’s major urban areas with high socioeconomic levels [35]. Eight of the studied provinces have a high proportion of older adults; these provinces are Yogyakarta, 15.52%; East Java, 14.53%; Central Java, 14.17%; North Sulawesi, 12.74%; Bali, 12.71%; South Sulawesi, 11.24%; Lampung, 10.22%; and West Java, 10.18% [11].

### Assessment of quality

**Fig. 3:**
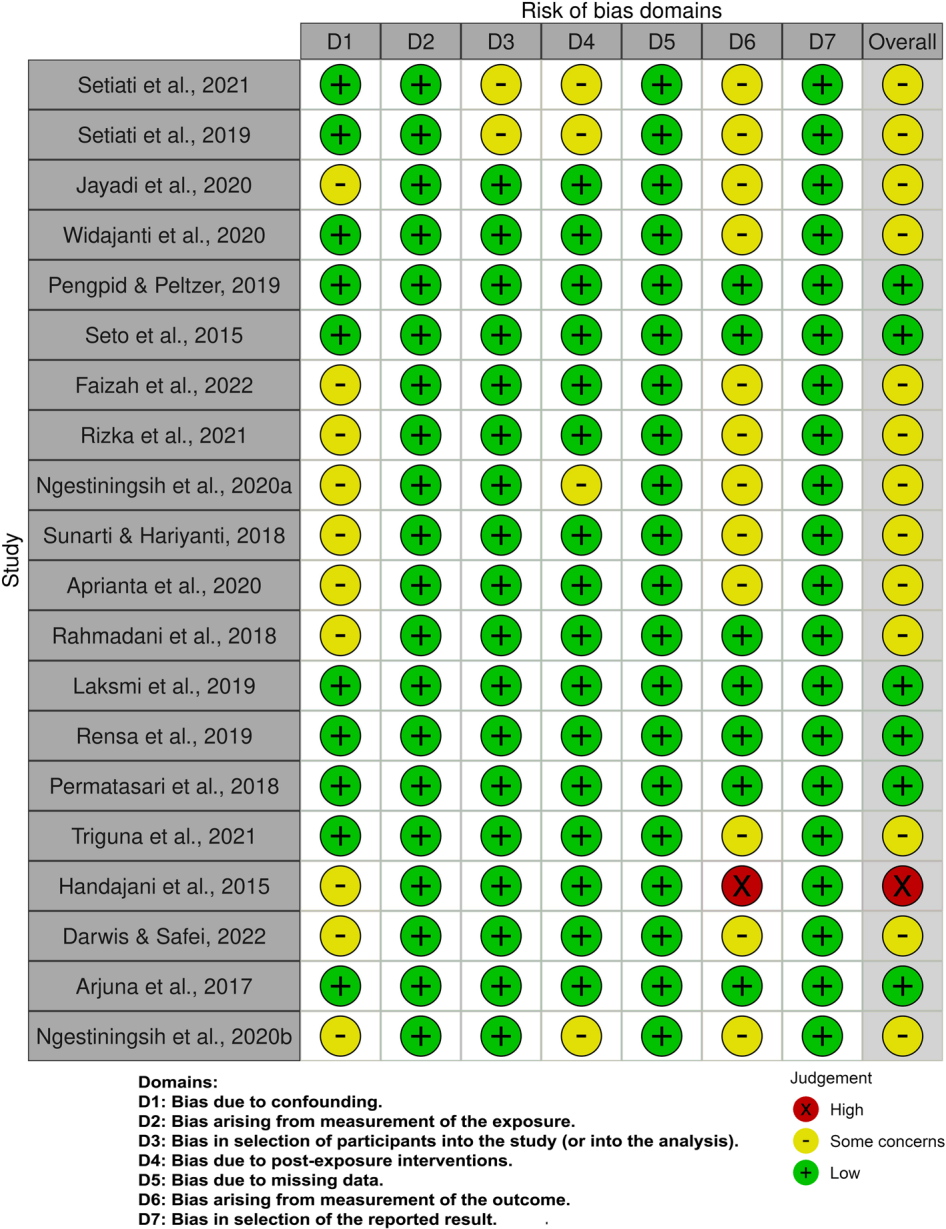
Risk of bias of included articles

An assessment of the study quality (Fig. 3) revealed that 6 (30%) studies had high quality, 13 (65%) had some concerns, and 1 (5%) had low quality (high risk of bias). In most articles, the effects of key confounders were not adjusted for.

### Pooled prevalence of frailty and prefrailty

**Fig. 4:**
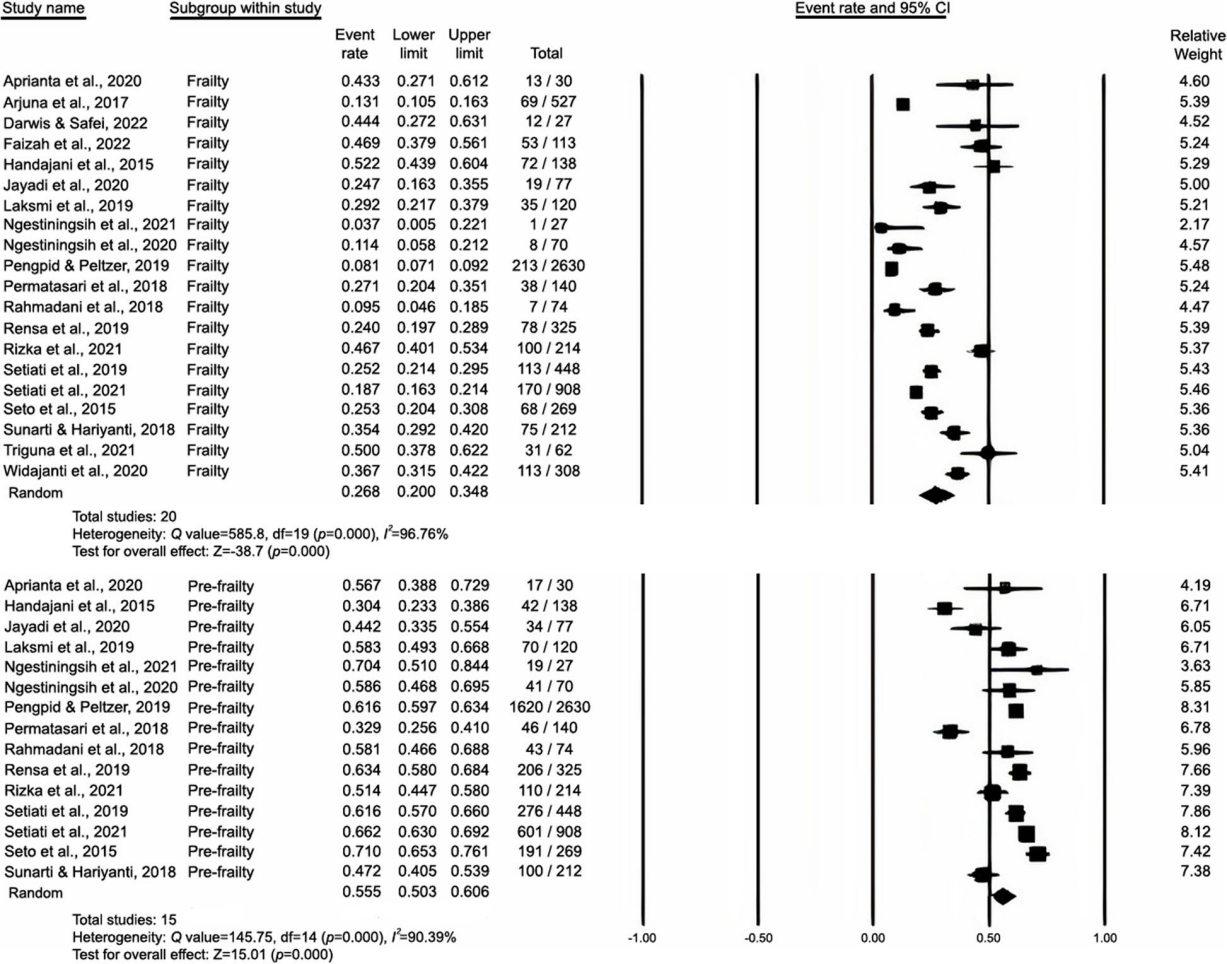
Prevalence of frailty and prefrailty in Indonesia

**Fig. 5:**
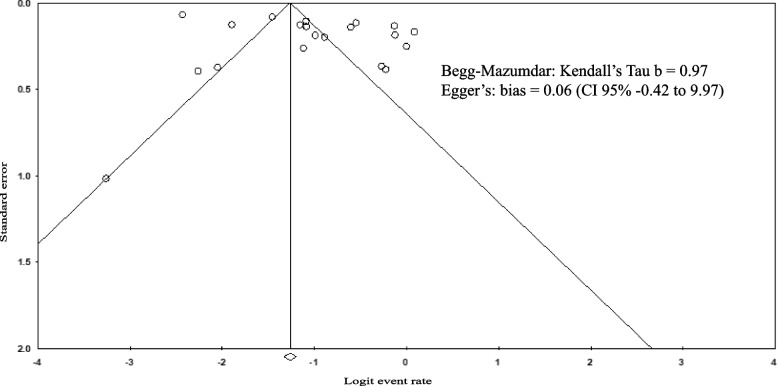
Funnel plot of included articles

Using a random-effects model, we performed meta-analyses for the prevalence of frailty and prefrailty, with 20 and 15 studies included, respectively. The prevalence of frailty ranged from 4.30% to 52.20%, whereas that of prefrailty ranged from 30.40% to 71%. As presented in Fig. 4, the pooled prevalence of frailty was 26.8% (95% CI: 20%–34.8%) and that of prefrailty was 55.5% in the Indonesian older adults (95% CI: 50.3%–60.6%). Publication bias was not detected (Fig. 5), and the results of the Begg-Mazumdar and Egger’s test were not significant (*p* = 0.9 and 0.06, respectively).

A high *I*^2^ value (*I*^2^ = 96.75%) was obtained, which indicated heterogeneity across the included studies. Therefore, sensitivity analysis, meta-regression, and subgroup analysis were performed for bias reduction. The sensitivity analysis revealed a similar prevalence of frailty (25.6%; CI = 19.1–33.4; *I*^2^ = 96.76, p < 0.001) and pre–frailty (57.5%; CI = 52.9–61.9; *I*^2^ = 90.39, p < 0.001) after one study with a high risk of bias [30] was excluded from the analysis. A meta-regression (Table 2) indicated that 1 year of data collection partly explained the heterogeneity observed in the prevalence of frailty (*ꞵ* = 0.33; *p* = 0.0007), whereas age (*ꞵ* = 0.07; *p* = 0.4257) and sample size (*ꞵ* =  − 0.0016; *p* = 0.1729) could not explain the heterogeneity. Sub-group analysis was conducted on two variables, namely measurement tools and study settings (Table 3).

**Table 2:** Meta-regression analysis results

Covariate	n	Coefficient	Std. Err	t	*p*	95% Conf. Interval	
Age	11	0.0745	0.0936	0.80	0.4257	-0.1088	0.2579
Sample Size	10	-0.0016	0.0012	-1.36	0.1729	-0.0039	0.0007
Year of Study	10	0.3346	0.0982	3.41	< 0.001	0.1421	0.5271

**Table 3:** Sub-group analysis results

Variable	Sub-variable	n	Prevalence% (95% CI)	*I*^2^ (%)	*p* value	Sample size
**Frailty**						
Measurement tools	FI-40	3	21.6 (15.5-29.2)	75.99	0.016	188/791
	FRAIL	3	18.7 (13.1-25.9)	87.92	< 0.001	277/1575
	Fried Phenotype	12	27.8 (17.2-41.6)	97.67	< 0.001	739/4188
Study settings	Community	8	21.1 (11.5-35.6)	97.67	< 0.001	528/3966
	Hospital	7	26.3 (21.4-32)	85.79	< 0.001	556/2270
	Nursing home	5	37.9 (25.2-52.6)	86.63	< 0.001	204/483
**Prefrailty**						
Measurement tools	FI-40	3	64.3 (56.5-71.4)	74.58	0.020	510/791
	FRAIL	2	49.7 (20.3-79.4)	98.06	< 0.001	647/1048
	Fried Phenotype	9	56.4 (51.3-61.4)	76.70	< 0.001	2217/3705
Study settings	Community	6	59.6 (55-64.1)	73.67	0.002	2262/3712
	Hospital	4	61.4 (52.4-69.6)	85.69	< 0.001	896/1374
	Nursing home	5	44.8 (33.5-56.7)	96.38	< 0.001	258/596

### Prevalence of frailty and prefrailty in studies stratified by measurement tools

**Fig. 6:**
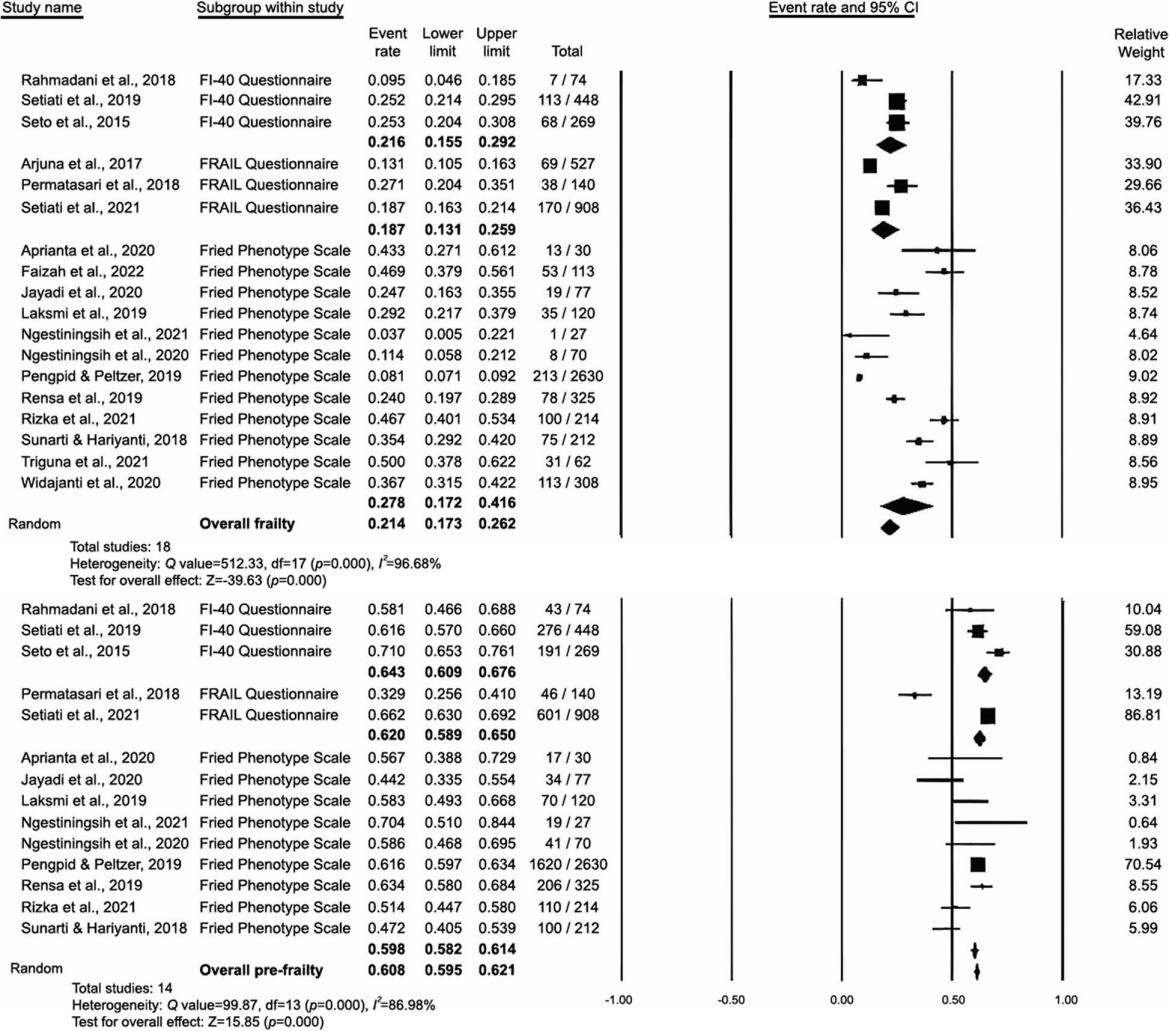
Measurement tools analysis of studies on the prevalence of frailty and prefrailty in Indonesia

Because the EFS and SHARE databases were used in only one study each, the sample size was insufficient for meta-analysis. The remaining 18 studies were included in a subgroup analysis stratified by measurement tools. The results revealed that the prevalence of frailty and prefrailty in studies using the FI-40 questionnaire was 21.6% (95% CI: 15.5%–29.2%) and 64.3% (95% CI: 60.9%–67.6%), that in those using the FRAIL questionnaire was 18.7% (95% CI: 13.1%–25.9%) and 62% (95% CI: 58.9%–65%), and that in those using the Fried Frailty Phenotype questionnaire was 27.8% (95% CI: 17.2%–41.6%) and 59.8% (95% CI: 58.2%–61.4%), respectively (Fig. 6). However, no significant difference was noted across measurement tools for frailty (*p* = 0.403) or prefrailty (*p* = 0.214).

### Prevalence of frailty and prefrailty in studies stratified by study settings

**Fig. 7:**
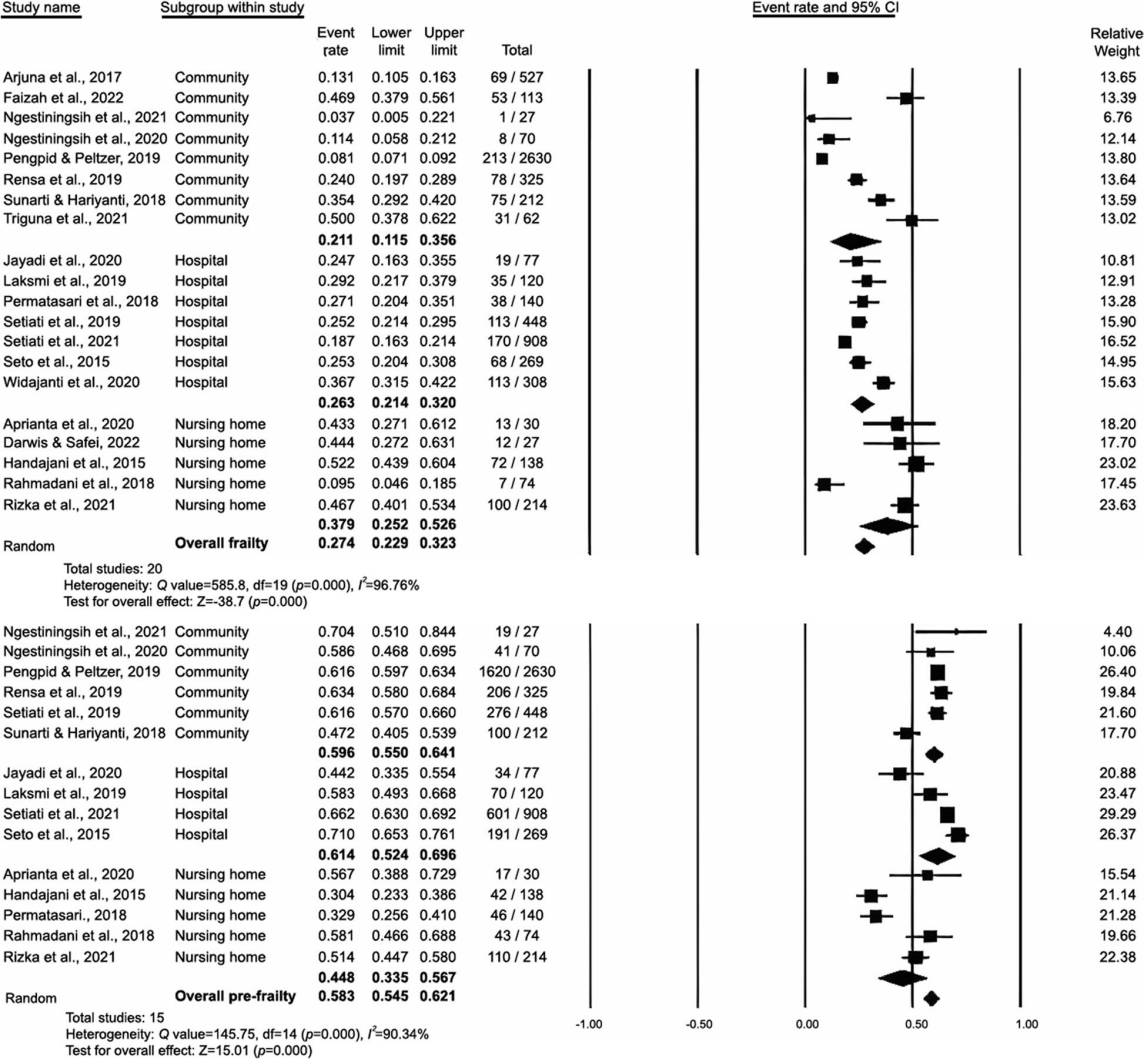
Study settings analysis of studies on the prevalence of frailty and prefrailty in Indonesia

The prevalence of frailty and prefrailty in studies conducted in nursing homes was 37.9% (95% CI: 25.2%–52.6%) and 44.8% (95% CI: 33.5%–56.7%), that in those conducted in hospitals was 26.3% (95% CI: 21.4%–32%) and 61.4% (95% CI: 52.4%–69.6%), and that in those conducted in community settings was 21.1% (95% CI: 11.5%–35.6%) and 59.6% (95% CI: 55%–64.1%), respectively (Fig. 7). However, no significant difference was noted across study settings for frailty (*p* = 0.173) or prefrailty (*p* = 0.057).

## Discussion

In this systematic review and meta-analysis, we investigated the overall prevalence of frailty and prefrailty in Indonesia. Our findings reveal that the pooled prevalence of frailty and prefrailty in Indonesian older adults was 26.8% and 55.5%, respectively. Stratified analysis revealed that the prevalence of frailty and prefrailty was 37.9% and 44.8%, 26.3% and 61.4%, and 21.1% and 59.6%, respectively, in studies conducted in nursing homes, hospitals, and community settings, respectively. The prevalence of frailty and prefrailty was 21.6% and 64.3%, 18.7% and 62%, and 27.8% and 59.8%, respectively, in studies using the FI-40, FRAIL, and Fried Frailty Phenotype questionnaire, respectively. However, no significant difference was observed across settings or measurement tools.

The lowest and highest prevalence was 4.30% and 52.20% for frailty and 30.40% and 71% for prefrailty, respectively. The reasons for the observed heterogeneity across the studies may be associated with differences in the study settings, population characteristics, and measurement tools. Information on the accurate prevalence of frailty is crucial for researchers involved in frailty research. Although Indonesia has a large population, the quality of its health services and the healthiness of the average person’s lifestyle in the country are not more favorable than those of developing or developed countries. Heterogeneity across regions in terms of health service distribution and a low level of health-related awareness among Indonesian individuals are the primary factors that have led to the country having a high rate of health problems, such as frailty [11].

The prevalence of frailty varies with the measurement tool used. Specifically, the multidimensional frailty tool (such as FI-40) provided a higher estimate of frailty than the physical dimensional frailty tool (such as Fried frailty phenotype and the FRAIL questionnaire) [4, 46–48]. However, we discovered no significant between-study differences in the results of measurement tools used for evaluating frailty and prefrailty prevalence in Indonesian older adults. Our data indicated a high prevalence of frailty assessed using the Fried Frailty Phenotype and different estimates of frailty prevalence from the two physical dimensional frailty tools (Fried frailty phenotype 27% vs FRAIL quesionnaire18%). These results are unexpected but are likely explained by the limited number of studies using the FI-40 and FRAIL scale in this review to define frailty.

The lowest, most moderate, and highest pooled prevalence of frailty was observed in community settings, hospitals, and nursing homes, respectively (21.1%, 26.3%, and 37.9%, respectively), the difference among the settings was nonsignificant; such an order of the prevalence of frailty in different study settings was also observed in a South American study (23.0%, 39.1%, and 55.8% in community settings, hospitals, and nursing homes, respectively; [49]. Studies have indicated that older adults recruited from hospitals and nursing homes had higher frailty levels than did those recruited from communities. For example, the prevalence of frailty in community-dwelling older adults has been reported to be 4%–59% [50, 51], that in hospital inpatients has been reported to be 20%–47.4% [52, 53], and that in nursing home residents has been reported to be 1.7%–76.3% [54, 55]. The older adults recruited from these institutions have various chronic medical health problems that affect their daily lives and lead to frailty [55]. Frailty in patients with various diseases can increase treatment costs [52].

The lowest, most moderate, and highest prevalence of prefrailty was 44.8% (nursing homes), 59.6% (community settings), and 61.4% (hospitals). Although no significant difference was discovered among the settings, such an order was also observed in a study conducted in South America, with the study reporting a prevalence of 29.8% in nursing homes, 47.6% in community settings, and 50.7% in hospitals [49]. The prevalence of prefrailty was the lowest in nursing homes, which might be because of the availability of adequate nursing home care for older adults in Indonesia and the fact that older adults tend to cohabitate with family rather than live in a nursing home or hospital. Notably, the pooled (all study settings) prevalence of prefrailty was higher in the present study than that reported in other studies, with other studies reporting a prevalence of 35% in nursing homes [54], 47.3% in community settings [50], and 25.8%–36.4% in hospitals [53, 55]. The high prevalence of prefrailty in Indonesia is concerning because it is an early but reversible sign of frailty, which can lead to negative health outcomes.

Most of the included studies were conducted in the urban areas of Indonesia. Older adults residing in urban areas often face challenges that may contribute to a disadvantaged status. They have inadequate access to opportunities for physical activity and limited social support from their families and communities [51, 56]. This observation is different from the prevailing notion and may be explained by the following reasons. First, Indonesian urban residents tend to be individualistic and spend most of their time working. Thus, older adults do not receive adequate family support. Second, urban developments in Indonesia are not centered on providing social facilities, such as parks and welfare agencies. Moreover, public transportation facilities fail to address the needs of older adults. Third, the complicated referral procedure and health services of Indonesia’s national health insurance systems and limited health-care facilities in urban areas are key barriers to accessing adequate support as well as social and health facilities for older adults. Similar findings have been reported by a study conducted in China [57].

The strength of the present study lies in the fact that we comprehensively searched six electronic databases to identify relevant regional articles. No date or language restriction was imposed during the literature search. Our study has some limitations. First, limited data are available regarding the prevalence of frailty and prefrailty in older adults residing in several Indonesian provinces, particularly those in the countryside, where the levels of frailty and prefrailty may be different from those in urban areas. This may introduce a bias in the estimation of the overall frailty burden in Indonesia. Second, the quality of study methodology was high in only 30% of the included studies. Some concerns were noted for the remaining studies; in fact, one study had a high level of bias. Third, some of the included studies had a small sample size (< 30 participants in 3 studies), which might not have been representative of the local populations of older adults with frailty. Finally, not all measurement tools have the category of prefrailty; however, determining the prefrailty status is crucial because a large proportion of older adults who are prefrail are likely to become frail; timely identification of this status can lead to timely intervention and potential recovery.

## Conclusions

To the best of our knowledge, this study is the first meta-analysis to report the overall prevalence of frailty and prefrailty in older adults residing in Indonesia. The pooled prevalence of frailty and prefrailty in our study cohort was 26.8% and 55.5%, respectively. The prevalence did not vary significantly across study settings or measurement tools. The gradual increase in the number of older adults with frailty or prefrailty in Indonesia demands attention from the government, private sectors, health-care professionals, and the community. To address this problem, all stakeholders should jointly design effective strategies and policies by adopting a local cultural approach and enhancing the early detection and prevention of frailty and prefrailty in older adults residing in Indonesia.

## Data Availability

The datasets used and/or analyses during the current study are available from the corresponding author on reasonable request.

## Abbreviations

CIs: Confidence intervals
FI-40: Frailty Index 40
FRAIL: Fatigue, Resistance, Aerobic, Illness, and Loss of weight
PRISMA: Preferred Reporting Items for Systematic Reviews and Meta-Analysis
SD: Standard deviation
SHARE: Survey of Health, Ageing and Retirement in Europe
